# The gastric caeca of pentatomids as a house for actinomycetes

**DOI:** 10.1186/1471-2180-12-101

**Published:** 2012-06-08

**Authors:** Tiago D Zucchi, Simone S Prado, Fernando L Cônsoli

**Affiliations:** 1 Lab de Microbiologia Ambiental, EMBRAPA Meio Ambiente, Rodovia SP 340, Km 127,5, CP 69, 13820-000, Jaguariúna, SP, Brazil; 2 Lab de Interações em Insetos, Depto de Entomologia & Acarologia, Escola Superior de Agricultura “Luiz de Queiroz”, Universidade de São Paulo (ESALQ/USP), Av Pádua Dias 11, CP 9, 13418-900, Piracicaba, SP, Brazil; 3 Lab de Quarentena “Costa Lima”, EMBRAPA Meio Ambiente, Rodovia SP 340, Km 127,5, CP 69, 13820-000, Jaguariúna, SP, Brazil

**Keywords:** *Actinobacteria*, Bacterial diversity, Pentatomidae, Symbiosis

## Abstract

**Background:**

Microbes are extensively associated with insects, playing key roles in insect defense, nutrition and reproduction. Most of the associations reported involve *Proteobacteria*. Despite the fact that *Actinobacteria* associated with insects were shown to produce antibiotic barriers against pathogens to the hosts or to their food and nutrients, there are few studies focusing on their association with insects. Thus, we surveyed the *Actinobacteria* diversity on a specific region of the midgut of seven species of stinkbugs (Hemiptera: Pentatomidae) known to carry a diversity of symbiotically-associated *Proteobacteria*.

**Results:**

A total of 34 phylotypes were placed in 11 different *Actinobacteria* families. *Dichelops melacanthus* held the highest diversity with six actinobacteria families represented by nine phylotypes. *Thyanta perditor* (*n* = 7), *Edessa meditabunda* (*n* = 5), *Loxa deducta* (*n* = 4) and *Pellaea stictica* (*n* = 3) were all associated with three families. *Piezodorus guildini* (*n* = 3) and *Nezara viridula* (*n* = 3) had the lowest diversity, being associated with two (*Propionibacteriaceae* and *Mycobacteriaceae*) and one (*Streptomyceataceae*) families, respectively. *Corynebacteriaceae* and *Mycobacteriaceae* were the most common families with phylotypes from three different insect species each one*.*

**Conclusions:**

Many phylotypes shared a low 16S rRNA gene similarity with their closest type strains and formed new phyletic lines on the periphery of several genera. This is a strong indicative that stinkbug caeca can harbor new species of actinobacteria, which might be derived from specific associations with the species of stinkbugs studied. Although the well-known role of actinobacteria as a source of biomolecules, the ecological features of these symbionts on the stinkbugs biology remain unknown.

## Background

Insects are by far the most diverse and largest cosmopolitan group of existing living animals with over a million described species [1,2]. Their successful worldwide dissemination was largely influenced by their associations with microbes (mostly bacteria), which allowed insects to exploit nutritionally-deficient food sources, either by complementing the diet with essential nutrients (e.g., *Buchnera aphidicola* in aphids) [3] and/or aiding in food digestion (bacteria and protozoa in termites) [4]. However, some associations are not beneficial to the host and the bacteria can play a pathogenic role affecting the host fitness (reduced reproduction and longevity, and increased mortality) [5]. The interactions insect-microorganism had been mostly investigated focusing on entomopathogens (virus, bacteria and fungi), but the limitations to the study of secondary and primary symbionts due to the difficulties to culture them *in vitro* have been recently overcome. The development of molecular tools and the use of new technologies for metabolite analysis are allowing for in depth investigations on the interactions bacteria and insects develop [6,7].

Bacterial mutualists have been firstly studied for their ecological appeal on insect development, but have recently gained a lot of attention due to their exploitation for insect and/or insect-vectored disease control, either through their direct elimination [8] or paratransgenesis [9]. Although the promising advances which may arise by these techniques, the use of the most intrinsic association between insects and bacteria, i.e. obligatory endocellular symbionts, is still thoroughly untapped mainly because these symbionts are difficult to cultivate or are not cultivable yet, which implies on an extra effort to obtain positive results. On the other hand, secondary symbionts are relatively straight forward to isolate and may therefore become a breakthrough tool on biological control of insect pests. However, most of these bacteria establish loosen relationships with their hosts and efforts must be driven to identify the most persistent secondary symbiont species which colonize the insect.

Stinkbugs (Hemiptera: Pentatomidae) are widely distributed around the globe and many species are considered as agricultural pests. A particular region of their midgut, the gastric caeca, has been scrutinized due to its association with a community of bacteria and the possible role this microbiota may have on host nutrition [10]. Several pentatomid species had their midgut symbionts investigated by culture-independent approaches and the most abundant bacterial species were identified as belonging to the *Enterobacteriaceae*[11-13]. However, these studies have used universal primers and PCR conditions that would favor this particular group of bacteria, while bacteria that would require particular PCR conditions for successful amplification would not be detected or be a minor representative in the population of amplicons produced. These bacteria could also be key players in the process of symbiosis and have an important impact in host fitness.

Our observations of scanning electron micrograph images of the gastric caeca of species of stinkbugs indicated the existence of cells with a morphology that resembled that of *Actinobacteria* (data not shown). Actinobacteria are known to not amplify well in PCR conditions normally used employing the universal primers developed based on *Escherichia coli*, and it has already been reported associated with the gut of several orders of insects [14-17], including a couple of species belonging to Hemiptera-Heteroptera [18,19]. Despite the existent data on the nutritional contribution of gut-associated *Actinobacteria*[18], and the provision of an antibiotic-barrier against pathogens by actinobacteria associated with the host body surface [20,21], little is known on the diversity of *Actinobacteria* associated with the gut of insects [22].

Therefore, due to the lack of information on the actinobacterial diversity associated with the gut of stinkbugs, we aimed to characterize the actinobacteria communities inhabiting the gastric caeca of the pentatomids *Dichelops melacanthus, Edessa meditabunda*, *Loxa deducta*, *Nezara viridula, Pellaea stictica, Piezodorus guildinii* and *Thyanta perditor*, by using a culture independent approach.

## Results

**Figure 1:**
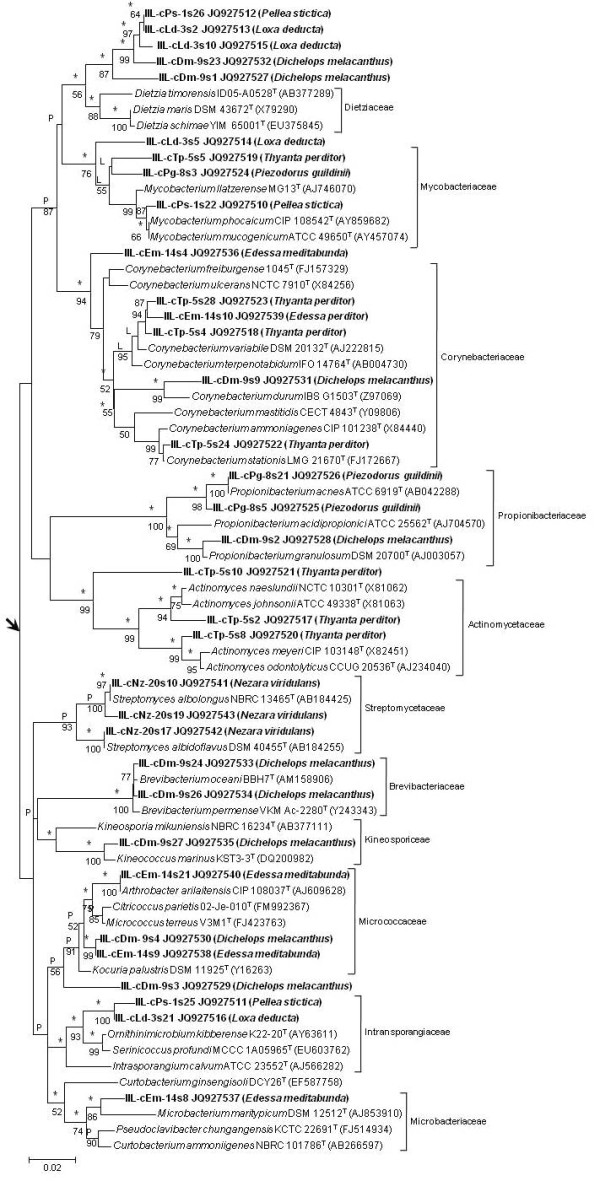
**Neighbour-joining tree based on 16S rRNA gene sequences (~640 bp) showing relationships between pentatomid gut-associated actinobacteria and closely free-living relatives.** Asterisks indicate branches of the tree that were also recovered with the maximum-likelihood and maximum-parsimony tree-making algorithm; L and P indicate branches which were either recovered with the maximum-likelihood or maximum-parsimony tree-making algorithm, respectively. Numbers at the nodes are percentage bootstrap values based on 1000 resampled data sets; only values above 50% are given. The arrow indicates the inferred root position using *Bacillus subtilis* DSM 10^T^ (GenBank accession no. AJ276351) and *Escherichia coli* ATCC 11775^T^ (X80725) which were used as the outgroup. Bar, 0.02 substitutions per nucleotide position.

The diversity of *Actinobacteria* associated with the V4 region of the midgut was quite different depending on the stinkbug species. *Dichelops melacanthus*, *T. perditor* and *E. meditabunda* had a quite diverse actinoflora associated, with several genera from different families of *Actinobacteria*. On the other hand, the actinoflora of *N. viridula* and *P. guildinii* were represented by one genus or a couple of genera from two distinct families, respectively (Table 1, Figure 1). Database search for sequence similarities to type strains ranged from 92.5 to 100% sequence identity (Table 1). In general, there is not a major, predominant phylotype within each stinkbug species. But *Mycobacteriaceae* are the most frequent whenever they occur (Table 1), with the exception of the phylotype of *Mycobacteriaceae* in *P. stictica*, which was almost as frequent as the others phylotypes.

**Table 1:** Nearest matches of 16S rRNA sequences (~640 bp long) of selected genotypes gut-associated actinobacteria from Pentatomidae

**Amplified from**	**Clones**	**Similarity with type-strain**		**%phylotype**^**a**^
		**Nearest match**	**Identity (%)**	
*Dichelops melacanthus*	IIL-cDm-9s1	*Dietzia maris* DSM 43672^T^ (X79290)	93.9	26.7
	IIL-cDm-9s2	*Propionibacterium granulosum* DSM 20700^T^ (AJ003057)	99.2	13.3
	IIL-cDm-9s3	*Citricoccus parietis* 02-Je-010^T^ (FM992367)	96.0	13.3
	IIL-cDm-9s4	*Citricoccus parietis* 02-Je-010^T^ (FM992367)	98.4	6.7
	IIL-cDm-9s9	*Corynebacterium durum* IBS G1503^T^ (Z97069)	97.2	6.7
	IIL-cDm-9s23	*Dietzia timorensis* ID05-A0528^T^ (AB377289)	95.5	6.7
	IIL-cDm-9s24	*Brevibacterium permense* VKM Ac-2280^T^ (AY243343)	99.5	6.7
	IIL-cDm-9s26	*Brevibacterium permense* VKM Ac-2280^T^ (AY243343)	99.5	13.3
	IIL-cDm-9s27	*Kineococcus marinus* KST3-3^T^ (DQ200982)	98.8	6.7
*Edessa meditabunda*	IIL-cEm-14s4	*Corynebacterium freiburgense* 1045^T^ (FJ157329)	97.3	6.3
	IIL-cEm-14s8	*Pseudoclavibacter chungangensis* CAU59^T^(FJ514934)	96.7	31.3
	IIL-cEm-14s9	*Citricoccus parietis* 02-Je-010^T^ (FM992367)	98.8	25.0
	IIL-cEm-14s10	*Corynebacterium variabile* DSM 20132^T^ (AJ222815)	98.3	25.0
	IIL-cEm-14s21	*Arthrobacter protophormiae* DSM 20168^T^ (X80745)	99.8	12.5
*Loxa deducta*	IIL-cLd-3s2	*Dietzia timorensis* ID05-A0528^T^ (AB377289)	95.9	37.5
	IIL-cLd-3s5	*Mycobacterium llatzerense* MG13^T^ (AJ746070)	95.6	50.0
	IIL-cLd-3s10	*Dietzia timorensis* ID05-A0528^T^ (AB377289)	95.5	6.3
	IIL-cLd-3s21	*Ornithinimicrobium kibberense* K22-20^T^ (AY636111)	97.3	6.3
*Nezara viridula*	IIL-cNv-20s10	*Streptomyces puniceus* NBRC 12811^T^ (AB184163)	100.0	20.0
	IIL-cNv-20s17	*Streptomyces violascens* ISP 5183^T^ (AY999737)	99.8	27.5
	IIL-cNv-20s19	*Streptomyces puniceus* NBRC 12811^T^ (AB184163)	98.4	52.5
*Pellaea stictica*	IIL-cPs-1s22	*Mycobacterium phocaicum* CIP 108542^T^ (AY859682)	99.2	25.0
	IIL-cPs-1s25	*Ornithinimicrobium kibberense* K22-20^T^ (AY636111)	96.5	37.5
	IIL-cPs-1s26	*Dietzia timorensis* ID05-A0528^T^ (AB377289)	95.9	37.5
*Piezodorus guildinii*	IIL-cPg-8s3	*Mycobacterium phocaicum* CIP 108542^T^ (AY859682)	96.6	73.3
	IIL-cPg-8s5	*Propionibacterium acnes* KPA171202^T^ (AE017283)	98.8	13.3
	IIL-cPg-8s21	*Propionibacterium acnes* KPA171202^T^ (AE017283)	99.8	13.3
*Thyanta perditor*	IIL-cTp-5s2	*Actinomyces naeslundii* NCTC 10301^T^ (X81062)	97.1	11.1
	IIL-cTp-5s4	*Corynebacterium variabile* DSM 20132^T^ (AJ222815)	98.6	5.6
	IIL-cTp-5s5	*Mycobacterium phocaicum* CIP 108542^T^ (AY859682)	96.4	44.4
	IIL-cTp-5s8	*Actinomyces meyeri* CIP 103148^T^ (X82451)	98.6	5.6
	IIL-cTp-5s10	*Curtobacterium ginsengisoli* DCY26^T^ (EF587758)	92.5	5.6
	IIL-cTp-5s24	*Corynebacterium stationis* LMG 21670^T^ (AJ620367)	99.4	11.1
	IIL-cTp-5s28	*Corynebacterium variabile* DSM 20132^T^ (AJ222815)	98.4	16.7

Similarities compared with entries from EzTaxon database. ^a^values corresponding to phylotypes obtained from each pentatomid species.

The phylogenetic analysis of the 16S rRNA gene placed all the 34 phylotypes from stinkbugs within the *Actinomycetales* order (Figure 1). In general, the analysis was suffice to determine the family of the phylotypes and 25 of them were distributed into 10 families: *Corynebacteriaceae* (*n* = 5 phylotypes); *Micrococcaceae, Mycobacteriaceae, Propionibacteriaceae* and *Streptomycetaceae* (*n* = 3 phylotypes each); *Actinomycetaceae*, *Brevibacteriaceae* and *Intransporangiaceae* (*n* = 2 phylotypes each); *Kineosporiaceae* and *Microbacteriaceae* (*n* = 1 phylotype each) (Figure 1). However, our results demonstrated that phylotypes which shared a 16S rRNA gene similarity value lower than 96.0% with their nearest type strain, although strongly associated with families included in the order *Actinomycetales*, formed new phyletic lines on the periphery of 16S rRNA gene subclade of known actinobacteria families. Therefore, it was not possible to assign them into a specific family. This was the case of IIL-cDm-9s1 which grouped together with other four phylotypes and formed a new 16S rRNA gene subclade closely associated with the subclade represented by sequences of the 16S rRNA gene of *Dietziaceae*. The two subclades were supported by all tree-making algorithms and by a bootstrap value of 56%. Similarly, the IIL-cDm-9s3, IIL-cLd-3s5 and IIL-cTp-5s10 phylotypes formed new phyletic lines strongly associated with *Micrococcaceae*, *Mycobacteriaceae* and *Actinomycetaceae* 16S rRNA gene subclades, respectively, with bootstrap supporting values from 56% to 99%. Furthermore, the highest phylotype diversity found for *D. melacanthus* was also represented by a high number of *Actinomycetales* families as this insect was associated with actinobacteria representatives scattered into five families and two other unresolved families (Figure 1). Similarly, the actinobacteria phylotypes from *T. perditor* were distributed into three families and one unresolved family, whereas *E. meditabunda* and *P. guildinii* had representatives within three and two families, respectively. *Loxa deducta* and *P. stictica* have actinobacteria representatives distributed into two families and one unresolved family. On the other hand, all phylotypes associated with *N. viridula* were comprised into a single family, *Streptomycetacea.*

## Discussion

The bacterial diversity associated with the midgut of stinkbugs has been investigated by a wide range of molecular analyses [5,11,23,24], but studies addressing the actinobacteria community within pentatomids have been thoroughly neglected. The present study is the first in which selective primers for actinobacteria have been applied to survey the diversity of this bacterial group into the gastric caeca of pentatomids (Hemiptera: Pentatomidae) and revealed a rich diversity of actinobacteria inhabiting their gastric caeca*.*

Actinobacteria are known inhabitants of the intestinal tract of several insects, but little has been reported on their role. Termites were shown to have a fixed community of actinobacteria mostly represented by species of *Actinomycetales*[25], and several of the *Streptomyces* species associated with the gut of termites have the potential to degrade hemicellulose and lignin, which are key components of the diet of these insects [14,26]. A very diverse community of actinobacteria, including species belonging to *Dietzia*, was also reported as gut inhabitants of scarabaeid beetles. These actinobacteria were also shown to release enzymes capable of degrading xylan and pectin as substrates [17,27]. Although these actinobacteria were show to produce a number of active enzymes that act on the food substrate of their hosts, their direct contribution to the digestive process and nutrition of their hosts has not been evaluated yet.

A number of associations among actinobacteria and hemipterans have also been reported, but far less diverse than those we report or those already described in termites and scarabaeids. *Coriobacterium glomerans* (*Coriobacteriaceae*) has been reported from the midgut of *Pyrrhocoris apterus* (Pyrrhocoridae) [28], and *Rhodococcus rhodnii* (*Nocardiaceae*) from the reduviids *Rhodnius prolixus, Rhodnius ecuadoriensis* and *Triatoma infestans*[29-31], and *Rhodococcus triatomae* from *Triatoma* sp. [32]. Although a horizontal transmission route for *C. glomerans* has been recently demonstrated and molecular analysis of another pyrrhocorid species indicated the occurrence of closely related species of actinobacteria [19], gut symbionts associated with *T. infestans* and *R. prolixus* were the only ones that have been shown to play a role in host nutrition by producing vitamin B [33,34].

We do not have sufficient information to argue on the role of the actinobacteria associated with the different species of stinkbugs we have studied in here. Nonetheless, it is striking how diverse the actinoflora associated with the gastric caeca of some of these stinkbugs are as compared to others, including the species of kissing bugs. However, the same pentatomids species surveyed herein were analyzed using universal primers [11], *unpub. data* and none of the clones retrieved were characterized as actinobacteria. Thus, it is clear that the use of specific primers enhanced the chance to detect this special bacterial group and has, therefore, opened the opportunity to better understand the evolutionary forces which may drive the interactions between bacteria and pentatomids. Mutualistic associations with microorganisms generally occur in insects that exploit nutrient-limited food sources, and it is quite common in blood or sap-sucking hemipterans [35,36]. Blood sucking hemipterans are known to carry symbionts associated with their gut that complement the vitamin B deficiency in their natural diet [33,34], while sap-sucking hemipterans are commonly associated with symbionts housed within bacteriocytes or bacteriomes [36,37]. But there is also a number of heteropterans (Hemiptera) that are not sap or blood suckers that carry symbiont bacteria associated to their gut that affect host fitness [38,39], but only one is known to be an actinobacterium [19,28].

Although the reported potential of gut actinobacteria to produce enzymes to possibly aid in food processing by their hosts (termites and scarabaeids) or to synthesize nutrients (hemipterans), the well-known potential of *Actinobacteria* to produce bioactive metabolites has led some to argue that these bacteria may also have a more general role in host protection against the invasion of pathogenic bacteria [22]. This hypothesis has gained support by the growing body of information on the association of actinobacteria with insects, in which actinobacteria are ectopically associated with the integument of Hymenoptera to produce a plethora of antibiotics to protect their hosts or the host’s food source [7,20,21,40]. Insect symbiosis have been reported more than half a century ago [35] and has regained attention due to the possible exploitation of symbionts for insect pest and/or insect-vectored disease control [8,30,41] and the impact they can have on pest- and disease-control programmes [42]. However, the biotechnological potential of bacterial symbionts associated to insects is another face of insect symbioses that is seldom explored, especially the extracellular bacterial symbionts [40,41,43,44].

Furthermore, most of the genera found inhabiting the midgut of the pentatomids in here studied has already been reported associated with other insects. Some of them have a beneficial impact on the insect fitness, i.e., streptomycetes in hymenopterans [20,21] and corynebacterial symbionts in *Rhodnius* spp. [30]. Other genera, such as *Dietzia*[27,45] and *Brevibacterium*[46], have been recently isolated from insects and the last may play a pathogenic association with their hosts [47]. The ecological features of these interactions could be achieved by selective isolation of the symbionts. However, our initial attempts to culture the actinobacteria associated with a couple of the stinkbugs we have studied by using several selective media for actinobacteria (data not shown) were fruitless so far, indicating a likely intrinsic coevolutionary relationship between these organisms or the environment (insect midgut) have selected actinobacteria species that may require special nutritional requirements.

## Conclusions

Thus, it is clear that the gastric caeca of pentatomids can be considered as an untapped reservoir of putative new species of actinobacteria. The new 16S rRNA gene subclade formed by the IIL-cDm-9s1 phylotype justifies any attempt to isolate and cultivate the actinoflora associated to stinkbugs. Finally, although many have sought to characterize the microbiological diversity in the stinkbug midgut, the simple use of a different primer set demonstrated the existence of a high diversity of an earlier unnoticed group of bacteria, indicating that the interactions between these insects and their symbionts are more complex than previously thought.

## Methods

### Insect sampling and midgut extraction

The pentatomids *Dichelops melacanthus*, *Nezara viridula*, *Edessa meditabunda*, *Loxa deducta*, *Pellaea stictica, Piezodorus guildinii* and *Thyanta perditor* were collected in a soybean field (South of Brazil, 2008/2009) and kept under laboratory conditions on a mixed diet composed of green beans and soybean and peanut seeds. From each studied species two adult females were surface sterilized before dissection. Their midguts were dissected under a microscope and transferred to a clean glass slide. The posterior section of the midgut (V4, crypt or caeca-bearing region) was removed, washed three times in sterile phosphate-buffer saline (PBS), macerated and then subjected for DNA extraction. Dissections were carried out under sterile conditions and all tools used were autoclaved before use.

### 16S rRNA gene sequencing analysis

The genomic DNA from the V4-midgut section of all individuals was extracted following Sunnucks and Hales [48]. The 16S rRNA gene was selectively amplified from purified genomic DNA by using primers designed for general identification of actinobacteria (S-C-Act-0235-a-S-20: 5′-GGCCTATCAGCTTGTTG-3′ and S-C-Act-0878-a-A-19: 5′-CCGTACTCCCCAGGCGGGG-3′) [49]. The polymerase chain reaction (PCR) mixture contained 10 ng gDNA, 1x PCR buffer, 1.5 mM MgCl_2_, 0.2 mM of each deoxyribonucleoside triphosphate, 0.32 μM of each primer, 0.5 U GoTaq polymerase, and sterile MilliQ H_2_O to 25μL. PCR condition used the touchdown protocol recommended by Stach et al. [49]. The PCR product was visualized by electrophoresis in a 0.8% (w/v) agarose gel, and the PCR product was purified using a PCR Product Purification Kit (Qiagen, USA), according to the manufacturer’s instructions.

The PCR product was then cloned into the pGEM-Teasy cloning vector and positive clones were selected following the manufacturer’s guidelines (Promega). Plasmids of selected clones (10 per individual, two rounds of 10 clones/pentatomid species) were extracted, purified and subjected to RFLP-PCR analysis prior to sequencing. Amplicons produced with the original primer set (S-C-Act-0235-a-S-20 and S-C-Act-0878-a-A-19) were subjected to restriction analysis with three informative restriction enzymes, *Eco*RI, *Msp*I and *Sal*I, and those which showed a different RFLP pattern were selected and sequenced using T7 and M13 universal primers. 16S rRNA gene sequences were compared with entries in the updated EzTaxon database [50]. The nucleotide sequences of 16S rRNA gene sequences of the phylotypes have been deposited with the GenBank database under accession numbers JQ927510–JQ927543.

### Phylogenetic analysis

Sequences were aligned using the MEGA4 software [51], and manually trimmed before further analysis. Phylogenetic trees were inferred by using the maximum-likelihood [52], maximum-parsimony [53] and neighbour-joining [54] tree-making algorithms drawn from the MEGA4 [51] and PHYML [55] packages. The Jukes and Cantor [56] model was used to generate evolutionary distance matrices for the neighbor-joining data. Topologies of the resultant trees were evaluated by bootstrap analysis [57] of the neighbour-joining method based upon 1,000 replicates using the MEGA4 software. *Bacillus subtilis* DSM 10^T^ (GenBank accession no. AJ276351) and *Escherichia coli* ATCC 11775^T^ (X80725) were used as outgroups.

## Authors’ contributions

TDZ, SSP and FLC planned the research. TDZ and SSP performed the cloning and RFLP analysis. TDZ carried out nucleotide sequencing and phylogenetic analysis. SSP collected the samples and revised the manuscript. TDZ and FLC wrote the manuscript. All authors read and approved the final manuscript.
